# A Cadaver Based Comparison of Two Elastic Tension Proximal Interphalangeal Joint (PIPJ) Extension Orthoses with Focus on Force Generation and Pressure Distribution

**DOI:** 10.3390/jcm12082855

**Published:** 2023-04-13

**Authors:** Vicenç Punsola-Izard, Nuria Carnicero, Elena Ozaes-Lara, Judit Mendieta-Zamora, Gemma Romera-Orfila, Karen S. Schultz, Manuel Llusà, Aroa Casado

**Affiliations:** 1 Hand Therapy Barcelona Physical Therapy and Clinical Investigation Center, 08010 Barcelona, Spain; nu.carnicero@gmail.com (N.C.); e.ozaes@gmail.com (E.O.-L.); judit.mendieta@eug.es (J.M.-Z.); gromeraorfila@gmail.com (G.R.-O.); aroa.casado@eug.es (A.C.); 2 Physiotherapy Department, Gimbernat School of Physical Therapy, 08174 Barcelona, Spain; 3 Karen Schultz Hand and Upper Limb Strategies (KSHULS), Littleton, CO 80120, USA; kshuls.cht@gmail.com; 4 Unit of Human Anatomy and Embryology, University of Barcelona, 08036 Barcelona, Spain; mllusa@ub.edu; 5 Department of Evolutionary Biology, Ecology and Environmental Sciences, University of Barcelona, 08007 Barcelona, Spain

**Keywords:** proximal interphalangeal joint, finger treatment, orthosis, hand therapy, flexion contracture, total end range time

## Abstract

Proximal interphalangeal joint flexion contracture is a frequent condition in hand therapy. Clinicians most frequently apply orthosis management for conservative treatment. Orthoses should apply forces for long periods of time following the total end range time (TERT) concept. These forces necessarily transmit through the skin; however, skin has physiological limitations determined by blood flow. Using three fresh frozen human cadavers, this study quantified and compared forces, skin contact surfaces and pressure of two finger orthoses, an elastic tension digital neoprene orthosis (ETDNO) and an LMB 501 orthosis. The study also investigated the effects of a new method of orthosis construction (serial ETDNO orthoses) that customizes forces to a specific finger position. We evaluated forces and contact surfaces for multiple ETDNO models tailored to the cadaver fingers in multiple PIP flexion positions. The results showed that the LMB 501 orthosis applied pressures beyond the recommended limits if applied for more than eight hours a day. This fact was the cause of time limited LMB orthosis application. This results also show that, at 30° of PIPJ flexion, straight ETDNOs created a mean pressure approaching the end of the recommended pressure limits. If the therapist modified the ETDNO design, the skin pressure decreased and reduced the risk of skin damage. With the results of this study, we concluded that for PIPJ flexion contracture, the upper limit of force application is 200 g (1.96 N). Forces beyond this amount would likely cause skin irritation and possibly skin injuries. This would cause a reduction in the daily TERT and limit outcomes.

## 1. Introduction

### 1.1. The Challenge of Proximal Interphalangeal Joint Flexion Contractures

Proximal interphalangeal joint (PIPJ) flexion contracture frequently occurs after hand trauma [1]. Clinicians consistently choose mobilizing orthoses as the means to achieve passive range of motion (PROM) goals [2]. The key elements for increasing PIPJ PROM are high time doses of low-load stress that are adequate to position the shortened tissue near the end of its currently available length [3,4,5]. If the therapist fails to consider and control all three variables (time, load quantity and optimum tissue length), the desired result will not occur.

Other important contracture management issues include the type of force generator and force distribution, as well as the overall design of the device including higher vs. lower profile or no profile [6,7]. Therapists can choose from various types of force generators: serial static, static progressive and elastic tension (often termed “dynamic” orthotic) [2,8]. No evidence exists to define a clear indication for a specific amount of force, orthotic design force generator or a program to use the device. However, all therapists agree that orthotic comfort ensures the highest degree of compliance with the treatment [9,10].

Sustained force application to contracted tissue has demonstrated a high level of effectiveness for increasing PROM [9,10]. Effective orthosis construction entails important principles, including increasing surface contact between the hand and the orthosis and generating a 90° force vector to create the most effective stress delivery [11,12,13]. The orthosis producer must consider specific issues with regard to finger and hand characteristics, including finger size and the potential for volume variation. The consistent consideration of these essential design factors will result in a comfortable orthosis. Ignoring any of these factors will lead to adverse side effects. The experience of pain often leads to orthosis removal that in turn temporarily or permanently reduces orthosis’ effectiveness [12,14].

It is difficult to know the exact force that the orthosis applies when the patient dons the orthosis. The force is linked to the joint position. With greater joint flexion, the orthosis force increases [12]. If the clinician applies the highest tolerable force at a specific angle and the contracture angle increases, this could cause a force increase and result in harm to the skin [15].

Brand reminds us that an orthosis applies force to the joint through the surface of the body. He writes, “Often the limits on the amount of force we can use are set more by what the skin can stand than by what the joint can accept”. Devices such as an orthosis can create an external stress that can result in skin ischemia [15]. The orthosis applied force coupled with the contact surface area for force application mediate this external stress or pressure. When using an orthosis for periods of less than eight hours, Paul Brand suggested a limit of 75 g/cm^2^ or 50 mm of Hg of skin pressure. He made this estimation based on the average amount of skin contact area under an orthosis. He added that if this force lasts for periods of more than eight hours, therapists should reduce the orthosis pressure to 50 g/cm^2^ or 33 mm of Hg [15].

Pressure application to the skin beyond 33 mmHg can diminish blood flow [15]. Blood circulatory pressure limits for both the lymphatic drainage pressure [16] and scar treatment [17] have the same parameters. Skin pressure beyond the therapeutic ranges can cause the lymphatic vessels to collapse, facilitating the presence of edema, ischemia and, finally, necrosis. While not new information, these pressure parameters have not translated into a common evaluation method in the orthotic literature. This is most likely because therapists currently lack the technology to determine the amount of surface pressure an orthosis generates. Currently, the literature describes orthosis-generated forces in grams rather than an amount of pressure. Clinicians have depended upon expert judgment and patient feedback to set the orthosis force. Signs and symptoms, such as edema, skin injuries, inflammation and pain [3,7,10,18], signal to the clinician that the orthosis requires force modification.

The literature focused on PIPJ flexion contractures has recommended different optimal levels of orthosis force. Some authors suggest forces in a lower range of 100 g (0.98 N) to 200 g (1.96 N) [14,15], while others endorse an optimal force in a higher range of 100 g (0.98 N) to 300 g (2.94 N) [12]. Most PIPJ flexion contracture series in the literature [8,9,10,18] describe forces around 200–250 g (1.96 N–2.45 N). However, no evidence to support any force parameters exists and, at the present time, these force ranges are just clinical recommendations.

The research has indicated that patients cannot wear commonly used ETOs generating 250 g (2.45 N) for more than 12 h a day. These studies failed to come to a specific conclusion regarding the ideal force dose an ETO should generate to achieve near 24 h of use. These authors state that is “it may not be clinically practical to expect patients to comply with a daily TERT beyond twelve to fourteen hours” [18].

In a recent study, Punsola-Izard et al. presented results regarding patients who were able to wear the orthosis for more than 20 h a day [19]. The study used a soft custom neoprene orthosis method inspired by the Banana Splint^TM^ [20], with a design specific to an individual patient [21]. Called the ETDNO /(Elastic Tension Digital Neoprene Orthosis), it consisted of a neoprene tube with a stretched axial dorsal strip that generated extension tension. Using this approach, the therapist customized the size of the tube to the size of the patient’s finger [21].

In prior studies, commonly used spring wire ETOs achieved 18° of improvement in 8 weeks [9]. No patient in these previous research populations achieved full extension at the PIPJ even after 4.5 months of orthosis use. However, Punsola et al. showed that an increase in duration of orthosis wear time to more than 20 h per day translated to improved outcomes [19]. The ETDNO preliminary study achieved a mean extension increase of 23.5° in three weeks. Some of them even achieved 0° of PIPJ extension.

**Figure 1:**
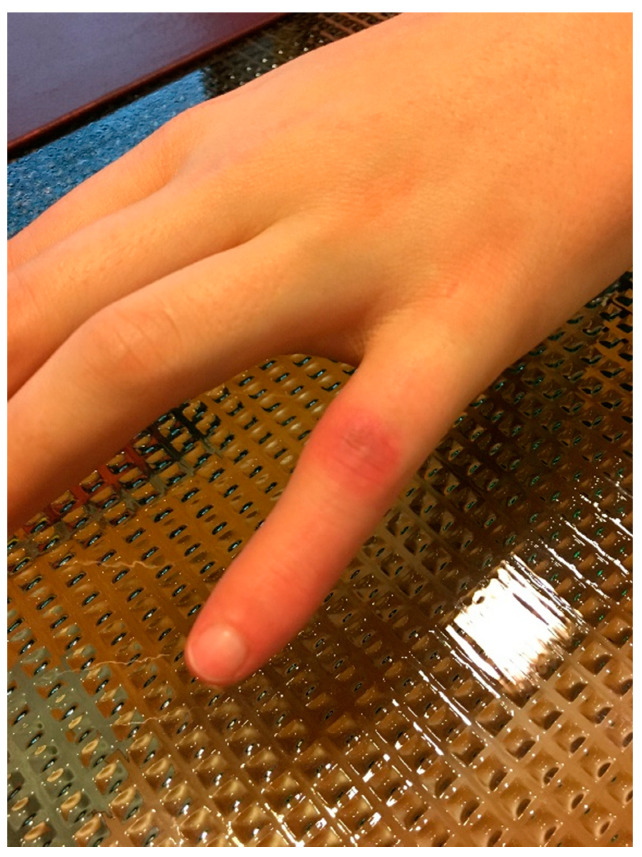
Sores at PIPJ.

The ETDNO study described a skin complication secondary to the orthosis use. This complication occurred when patients with a flexion contracture measuring greater than 30° received a straight tube ETDNO. This manifested in skin redness and sores on the dorsal aspect of the PIP joint. This pathology was a clear sign of the limit of skin tolerance to pressure [19] (Figure 1).

In a clinical case study, Punsola et al. demonstrated that a modification of the ETDNO tube shape for patients with more than 30° can treat the contracture without harming the skin [22]. While the original model of the ETDNO consisted of a straight tube, the modified ETDNO version used a bent tube adapted to the joint flexion contracture position. In this study, Punsola et al. described a protocol using a serial ETDNO procedure. This method sought to adapt the orthotic design—and so the force—to the specific joint position and change it when the flexion contracture improved by 10° [19].

### 1.2. Purposes of the Study

This study has three distinct purposes, which are divided into five hypotheses. The first component of this study is to quantify the forces in grams that the ETDNO and the LMB 501 PIP extension orthosis generate. The second component evaluates the contact surfaces of the ETDNO and the LMB 501 orthosis to understand the force distribution of both orthoses on the volar and dorsal aspect of the finger skin. Finally, the third purpose is to determine the pressure that both orthoses apply to the skin and compare this amount with the safe pressures described in the literature. The first purpose is related to the first, second and third hypothesis, while the second purpose is related to the fourth hypothesis and the third purpose to the fifth hypothesis.

### 1.3. Hypothesis

The study explored five hypotheses. The first hypothesis states that the LMB 501 orthosis generates higher forces than the ETDNO. The second hypothesis asserts that the straight tube ETDNO generates forces beyond the therapeutic range described by Brand (200 g) (1.96 N) when applied to the PIPJ with a contracture measuring 30° or more. The third hypothesis postulates that modification of the ETDNO tube shape will reduce the force at 30° of the PIPJ flexion to once again generate forces within the therapeutic range. The fourth hypothesis proposes that the ETDNO doubles the contact area of the finger skin when compared with the contact surface area of LMB 501 orthosis. Finally, the fifth hypothesis posits that the LMB 501 orthosis generates greater pressure on the dorsal and volar skin than the ETDNO.

## 2. Materials and Methods

To conduct these investigations, we compared a soft elastic tension custom orthosis, known as the ETDNO, with a popular commercial model of extension spring orthosis, known as the LMB 501 orthosis. We chose the LMB 501 orthosis because therapists can easily obtain it on the commercial market. This type of comparison has the potential to illustrate and possibly contrast the characteristics of the two orthoses.

**Figure 2:**
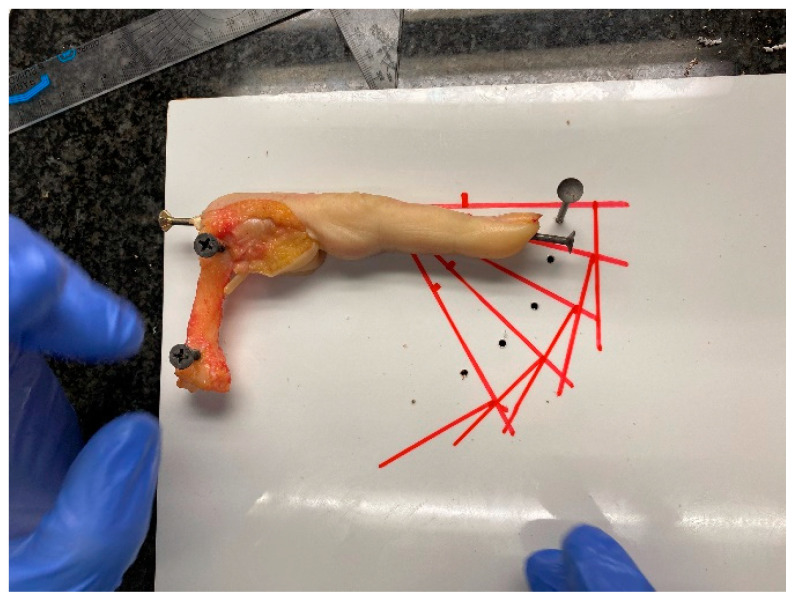
Anatomy lab evaluation model.

To address the first and second hypotheses of this study, which seek quantification of the orthoses force generation, the team prepared three fresh frozen cadaver finger models—a middle finger, a ring finger and a small finger—in the anatomy lab (Unit of Human Anatomy and Embryology, University of Barcelona) and mounted them on a wooden support. With the metacarpophalangeal joint stabilized in flexion and the DIP joint immobilized, only the PIP joint had free movement (Figure 2).

**Figure 3:**
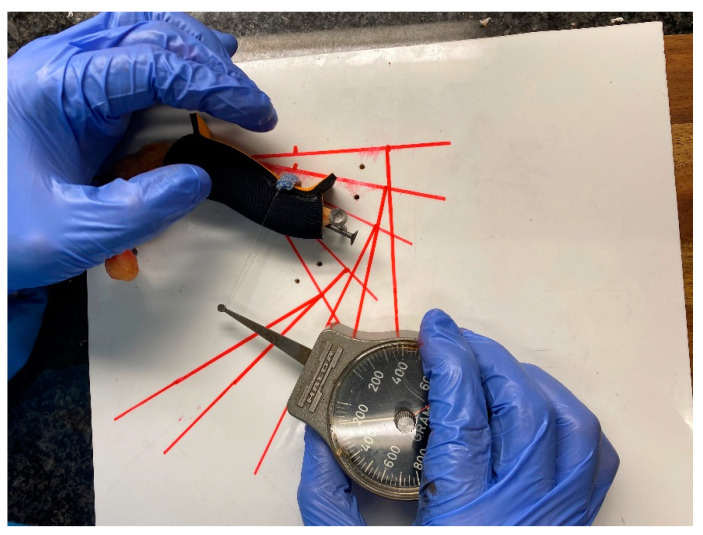
Measurement system of orthoses.

Researchers drew guide-lines on the wooden support to ensure that when applying the dynamometer, they applied force without varying the lever arm from the joint axis. This approach ensured that the force vector was always at 90° (Figure 3). To avoid measurement errors, three people performed and recorded all measurements three times to obtain the mean. This resulted in a total of nine measurements.

**Figure 4:**
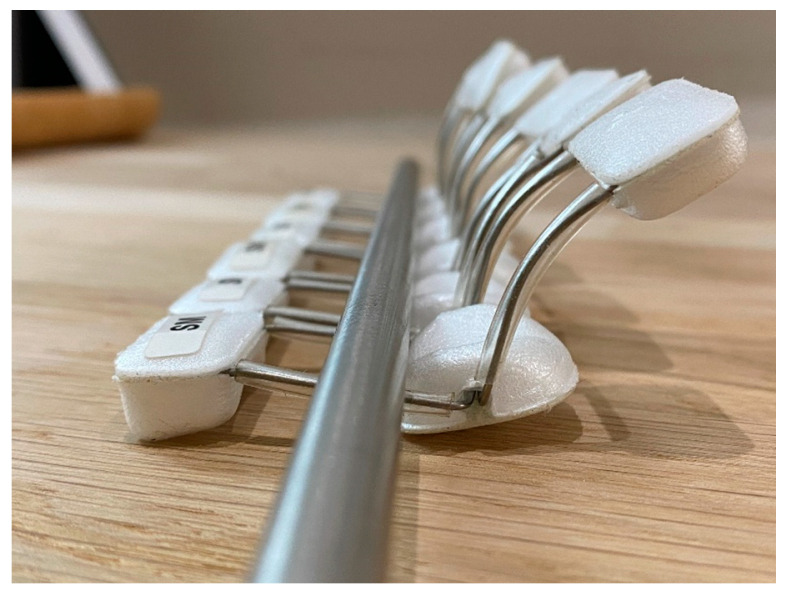
LMB 501 orthoses.

Researchers constructed 30 straight tube ETDNOs (ten for the long finger, ten for the ring finger and ten for a little finger). They also obtained 20 LMB 501 orthosis (ten medium size for the middle and ring finger and ten small size for the little finger) for testing (Figure 4).

To evaluate forces of the ETDNO and the LMB 501, the investigators applied each ETDNO and LMB 501 orthotic to each anatomical model. They measured the force for each orthotic three times at five different PIPJ flexion positions: 0°, 15°, 30°, 45° and 60°. They applied a traction system to the dorsal aspect of the head of the second phalanx. This traction was applied at 90° of the phalanx following the drawn guide-lines. A HALDEX gauge™ measured the force that the orthosis applied to the finger at each of these positions. We recorded these forces and obtained the mean (Figure 4). We compared the mean extension force of the ETDNO to the mean force of the LMB 501 of the same finger in every position. Following application of all the straight tube ETDNOs to all three fingers at 30°, we calculated the mean force of the straight tube ETDNO at 30°.

**Figure 5:**
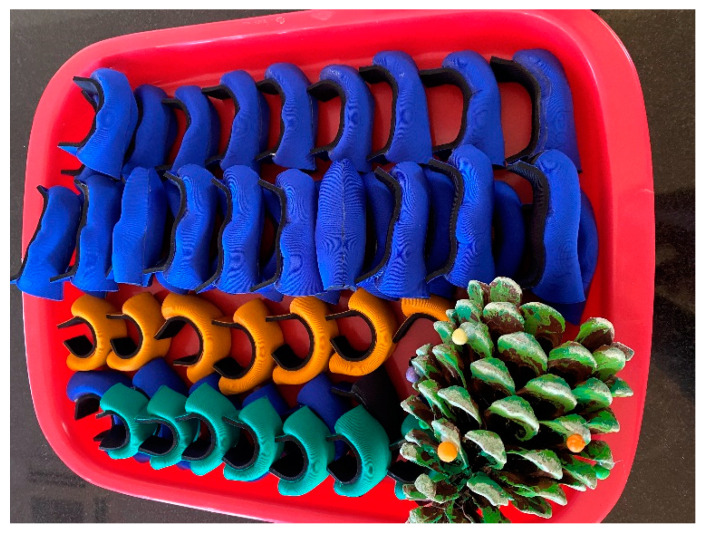
ETDNO with tubes at different positions.

To investigate the third hypothesis of the study, researchers constructed 50 ETDNOs. The ETDNOs consisted of five groups of ten ETDNOs. Each group had a different tube angle. The first group had ETDNO tubes hyperextended at 15°, the second group featured a straight tube, the third group had ETDNO tubes at 15° of flexion, the fourth group of ETDNO tubes had 30° flexion and, finally, the last group had ETDNO tubes at 45° (Figure 5). Only one therapist performed and recorded all measurements of the forces that the orthoses generated to obtain the mean force. We calculated the mean force of the ETDNO at +15°, 0°, 15°, 30° and 45°. We then compared all force-related computations to determine which ones were below the recommended level.

**Figure 6:**
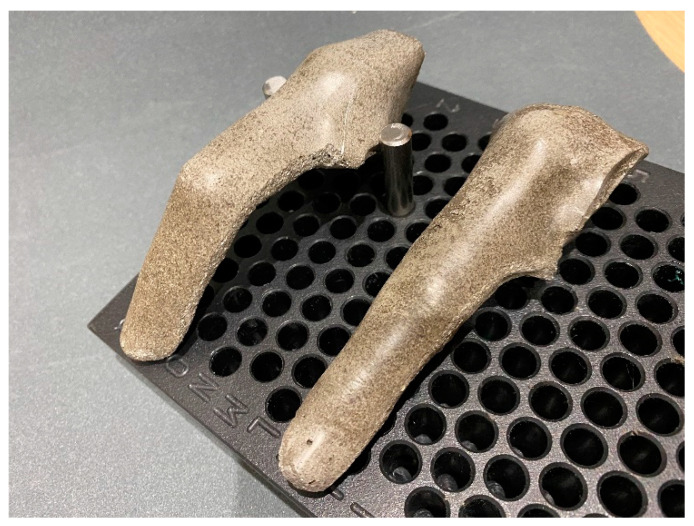
Concrete models.

**Figure 7:**
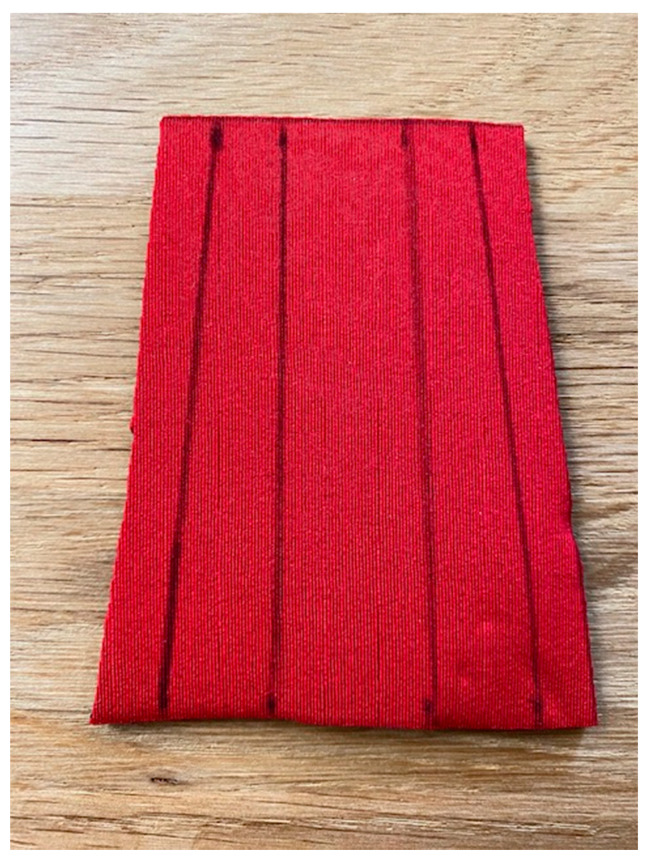
ETDNO pattern lines.

To evaluate the contact interface between the orthosis and the finger, the researchers first constructed two concrete models using the right-hand middle finger from the anatomy lab as a mold. The first concrete model featured a 30° flexion position of the PIPJ, while the second model postured 0° (Figure 6). This part of the study applied six LMB orthotics and 12 ETDNOs (six at 0° and 6 at 30°) to these models. Prior to construction of the tube for the ETDNO, the researchers drew lines on what would become the interior aspect of all ETDNO patterns (Figure 7). These lines divided the volar aspect of the finger from the lateral and medial aspects and, finally, the dorsal. This division helped us to determine the zones of the ETDNOs that actively contacted the finger surface and created an extension force.

**Figure 8:**
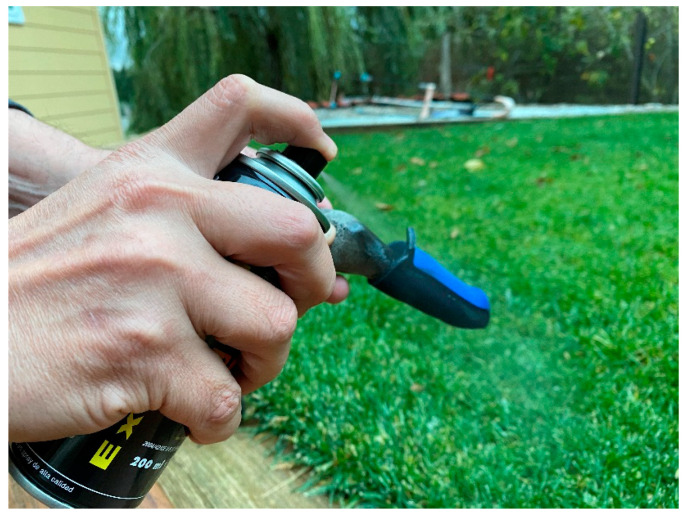
Spray application.

**Figure 9:**
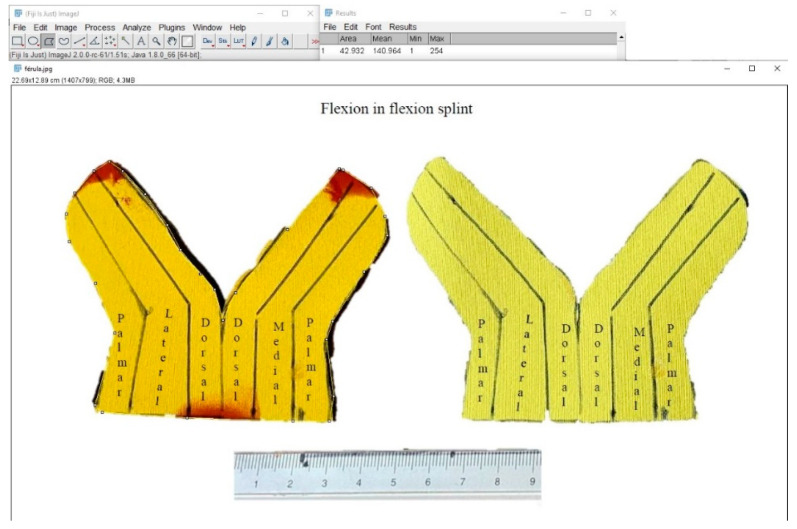
Measurement of superficial area in software Fiji-ImageJ.

We did not designate the contact areas of the LMB 501 because the finger volar and dorsal aspect contact areas were clear. After placement of the ETDNO orthoses on the concrete model, the researcher applied colored spray around the orthosis to clearly mark all the empty spaces between the orthosis and the finger (Figure 8). When the spray dried, the researchers removed the orthosis and examined the surfaces of the orthosis. Using the Fiji software (Figure 9), they measured the area of the original color that remained.

**Figure 10:**
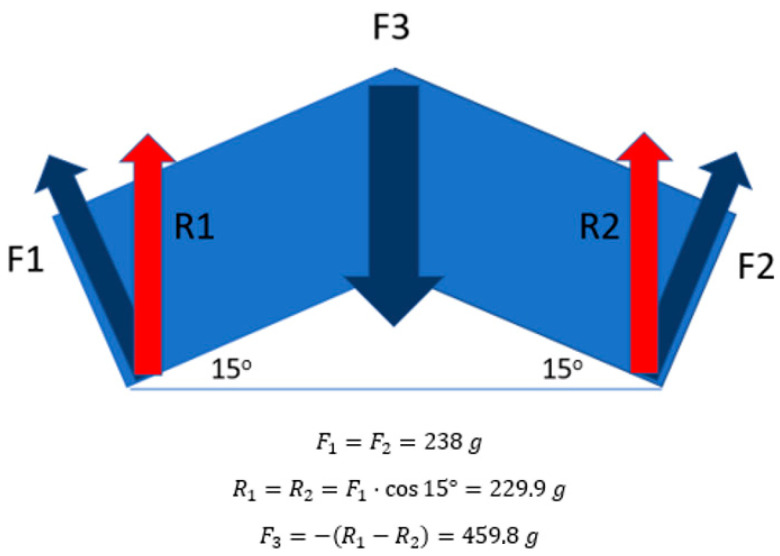
PIPJ dorsal force applied formula. When ETDNO is applied, it only covered the whole length of the second phalanx and 2/3 of the length of the lever arm of the first phalanx. We considered the distal and proximal lever arm of the PIPJ to be the same length. For this reason, we consider the distal and proximal forces applied to be of equal amounts. F1 and F2 were the forces that the ETDNO apply, while R1 and R2 was the results of the forces when considering the angle of the force. F3 was the force applied at the dorsal aspect of the PIPJ.

To aid in the evaluation of the ETDNO pressure distribution in the last hypothesis of the study, we combined force data obtained in the first part of the research and the contact surfaces data collected in the final part of the research. We then calculated the mean pressure that the straight tube ETDNO and the LMB 501 generated at 0° and at 30°. We chose these positions because we wanted to collect the data for mean pressure at both full extension and the position where skin problems occurred when using a straight tube ETDNO [22]. Finally, to determine if changing the orthosis design would reduce the pressures applied to the finger skin (Figure 10), we performed the same evaluation with a 30° flexed tube.

### Statistical Analysis

Statistical analyses were performed using Jamovi Stats for Windows (The Jamovi Project). We predicted the mean, standard error of the mean, standard deviation, 95% confidence interval and the kurtosis of the different measurements made for the mean extension force of the splints. To establish a comparison between the two study groups, we carried out a normality study of the sample using the Shapiro–Wilk statistical test. The statistical analysis corroborated the distribution of the sample. We then applied the non-parametric Mann–Whitney U statistical test to explore the group differences (Supplementary Tables S1–S3).

## 3. Results

All the ETDNO and LMB 501 orthotics tested in the study created an extension torque. The forces of all the orthoses increased proportionally as flexion in the PIP joint increased [12]. The data confirmed hypothesis one because all LMB 501 models were stronger than the ETDNO in all positions (Table 1).

At 30°, the mean extension force of the straight ETDNO was 238 g (standard deviation = 12.7, standard error of the mean = 4.03, 95% CI = 229–247, kurtosis −2.50) for the 3rd finger, 172 g (standard deviation = 14.2, standard error of the mean = 4.49, 95% CI = 162–183, kurtosis = 1.50) for the fourth finger and 215 g (standard deviation = 21.1, standard error of the mean = 6.67, 95% CI = 200–230, kurtosis = 0.37) for the fifth finger. The mean of the extension force of all three fingers force for the ETDNO was 208 g (standard deviation = 31.8, standard error of the mean= 5.81, 95% CI= 197–220, kurtosis = −1.18). The mean extension force of the LMB 501 at 30° was 282 g (standard deviation= 29.0, standard error of the mean = 9.17, 95% CI = 262–303, kurtosis = −1.23) for the 3rd finger, 245 g (standard deviation = 28.4, standard error of the mean = 8.98, 95% CI = 225–265, kurtosis = 0.55) for the fourth finger and 310 g (standard deviation= 47.4, standard error of the mean = 15.0, 95% CI = 276–344, kurtosis = 3.94) for the fifth finger. The mean of all three fingers force for the LMB 501 was 279 g (standard deviation = 44.1, standard error of the mean = 8.05, 95% CI = 263–275, kurtosis = 2.94). The data also confirmed the second hypothesis because the LMB 501 and the ETDNO force at 30° both exceeded 200 g (1.96 N), the upper limit of the therapeutic range indicated by Brand. The ETDNO exceeded the limit by 8 g, while LMB 501 exceeded the limit by 79 g (Table 1).

The modification of the shape of the ETDNO tube with respect to the finger joint position allowed a corresponding decrease in orthosis force. Considering Brand’s therapeutic range of force description, which lies between 100 g (0.98 N) and 200 g (1.96 N), we confirm the third hypothesis. The construction of the ETDNO with the tube in different positions generates forces within the therapeutic range when the ETDNO tube angles correspond to the different positions of the PIPJ (Table 2 and Figure 5).

In our investigation of the differences in contact area of the finger skin between the two orthoses, we first computed the total third finger skin surface area with a result of 51 cm^2^. We divided this surface into the volar, dorsal, medial and lateral surfaces. In total, 100% of the potential contact surface of the ETDNO of the dorsal and volar aspect of the finger measured 14.875 cm^2^, while the lateral and medial surfaces totaled 10.625 cm^2^. The volar finger contact surface of the ETDNO equaled 100% in any finger position and when using either the straight tube or the flexed tube. However, the dorsal contact surface pattern of the ETDNO changed with different PIPJ positions. Use of a straight tube ETDNO with the finger at 0° resulted in a dorsal contact area of 83%. With the finger flexed at 30°, this contact surface area reduced to 70%. Application of the 30° flexed ETDNO to 30° PIPJ increased the dorsal contact surface between ETDNO and the dorsal skin to 93%.

Contact patterns of the LMB 501 orthosis were similar whether the finger was at 0° or at 30°. The finger contact surface area at the volar aspect of the LMB 501 orthosis measured 38.31% with the PIPJ at 0° and 35.63% in flexion at 30°. On the finger dorsal aspect, the LMB 501 demonstrated a 17.5% contact surface area with the PIPJ at 0°. This contact area reduced to 16% in 30° of flexion. (Table 3). These findings refute the fourth hypothesis because the contact surface of the ETDNO was more than double that of the LMB 501. At the volar aspect of the finger, the ETDNO created 2.6 times the contact surface of the LMB 501. At the dorsal aspect of the finger, the ETDNO contact surface creates 4.3 times the contact surface of the LMB 501.

The researchers calculated the pressure on the volar and dorsal aspects of both the 0° and the 30° concrete finger models. When we applied the straight tube ETDNO to the 0° finger model, the pressure on the dorsal aspect of the model measured 5.97 mmHg This amount of pressure was well below the 33 mmHg limit. When we applied the same orthosis to the concrete model with the PIPJ at 30°, the pressure increased and achieved a mean of 32.43 mmHg. This figure clearly approached the recommended limits. When we applied the 30° Flexed tube ETDNO to the 30° PIPJ model, the pressure at the dorsal aspect of the finger was within the safe limits at 11.32 mmHg pressure. The flexed tube ETDNO successfully reduced pressure at the dorsal aspect of the finger.

**Figure 11:**
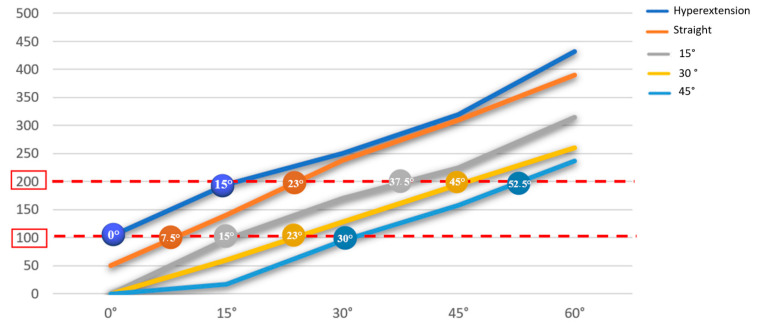
Therapeutic range of the ETDNO constructed at different angles. The values that are within the red range are the tolerable ranges for the patients in terms of pain.

Due to the reduced contact area, the LMB 501 orthosis exceeded the recommended pressure limits at the dorsal aspect of the finger in all cases. This occurred in the straight finger model and measured 43.57 mmHg, but also in the flexed finger where it generated a mean pressure of 166.96 mmHg. This latter measurement was five times the recommended limit. On the volar aspect of the finger, the LMB 501 orthosis generated safe pressure levels only if the PIPJ was at 0°. However, as soon as the PIPJ postured in flexion, pressures at the volar aspect also increased beyond the recommended limits. This data confirmed our last hypothesis (Table 4) (Figure 11).

## 4. Discussion

The treatment of flexion contracture of the proximal interphalangeal joint is a therapeutic challenge. To obtain the optimal result, one must find an optimal balance between maintaining the end range position and applying the ideal force for as long as possible. Any mistake involving excessive application of force can generate complications that result in an interruption in treatment and a reduction in daily TERT dose.

**Figure 12:**
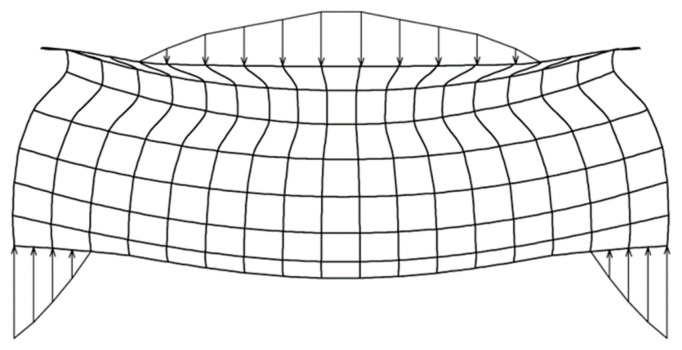
Diagram of ETDNO pressure distribution at dorsal and volar aspects of the finger.

In this study, we confirmed that the straight tube ETDNO generates a mean force of 208 g when applied to a 30° PIPJ flexion contracture. This force is too near Brand’s therapeutic range limit for an orthosis that a person wears for more than 8 h. While lower than the 250 g (2.45 N) that other authors used [9,10,18], these forces still translated into pressure gradients of 32.43 mmHg. These came very close to the 33 mmHg amount for orthosis wear longer than eight hours that Brand suggested as an upper pressure limit. By discovering that this value was a mean, we became aware that pressure can have higher peaks in some areas along the dorsal aspect of the PIPJ (Figure 12). The results of these studies support our agreement with Brand and Bell–Krotoski that therapists should maintain PIPJ orthoses forces at a therapeutic range below 200 g (1.96 N) [14,15].

The LMB 501 generates forces that extend beyond this safe therapeutic range [15] if worn for longer than 8 h and provably does so in even less time. We have shown that this level of force can create skin problems with orthotic treatments. Our measurements show that an LMB 501 generates force in the therapeutic range only between 5° and 20° of PIPJ flexion. If clinicians wish to use these orthoses with a PIPJ flexion contracture beyond 20°, they must also physically change the orthosis to adapt the force to a specific patient. They also need to constantly supervise the patient and, specifically, the patient’s finger skin. The clinician must instruct the patient to intermittently remove the orthosis to avoid problematic secondary effects. Previous studies of PIPJ flexion contracture that used commonly prescribed ETOs have concluded that patients will not be able to wear an orthosis more than 12 h.

The results of the present study demonstrate that as the orthosis design reduces the contact surfaces, pressure increases. The increase in pressure can cause a decrease in comfort. This could be the reason why continuous use of spring wire orthoses becomes impossible. When a clinician applies a spring wire orthosis to a PIPJ flexion contracture, this skin pressure can lead to intermittent use of the orthosis. This in turn causes a reduction in the daily TERT dose. The patient attempting to wear the commonly applied ETOs that have a small contact surface will likely have difficulty wearing the device for more that 12 h.

This research has shown how the modification of the ETDNO construction method to match the angle of the device to the angle of the PIPJ contracture reduces ETDNO force and increases the contact surface to between 70% and 93% of the dorsal skin. The reduction in force and increase in contact area combine to reduce orthotic pressure on the finger and so increases comfort. While some authors have concluded that it is impossible to wear an ETO for more than 12 h, Punsola-Izard leveraged the advantage of improved pressure distribution and lower force levels to achieve a mean of 20.5 h for orthosis wear time. This serial ETDNO protocol maintains the finger pressure within the therapeutic range that Brand recommended [15].

## 5. Conclusions

In this study, we have determined that the application of an appropriately designed ETDNO will generate maximum intensities below 200 g (1.96 N). We have also demonstrated that serially designed ETDNOs can enlarge the contact area between the orthotics and the finger, allowing a better distribution of forces and reducing the risk of pathologic levels of pressure. This data helps clinicians to understand the effectiveness of an ETDNO compared to other devices. These characteristics of lower forces distributed over a wider surface area clearly favor greater comfort and allow the patient to increase the TERT treatment dose. Finally, we have shown that the serial elastic tension orthosis method can apply the range of therapeutic forces to a wider range of finger positions because the therapist can construct new ETDNO models as the patient’s PIPJ angle changes. This orthosis sequence gives the patient an opportunity to avoid treatment interruptions and increase the daily TERT. Our research has demonstrated a means to optimize daily TERT and offer significantly improved outcomes for our patients. Using our sequential orthosis methodology, researchers should perform further studies to determine the correlation between lower forces and greater daily TERT dose in the treatment of PIPJ flexion contractures. This study is limited to the study of three fingers of a medium sized hand; if we consider that finger sizes can differ a lot from one patient to another, this study could be improved by studying a bigger sample of fingers.

## Figures and Tables

**Table 1:** Forces of straight tube ETDNO and LMB 501 orthosis for three different fingers and mean force of the orthosis at different positions.

	0°	15°	30°	45°	60°
ETDNO third finger	50 g (0.49 N)	140 g (1.37 N)	238 g (2.33 N)	310 g (3.04 N)	390 g (3.82 N)
ETDNO fourth finger	45 g (0.44 N)	110 g (1.08 N)	172 g (1.68 N)	237 g (2.32 N)	300 g (2.94 N)
ETDNO fifth finger	80 g (0.78 N)	158 g (1.55 N)	215 g (2.11 N)	275 g (2.70 N)	340 g (3.33 N)
MEAN ETDNO	58.33 g (0.57 N)	136.00 g (1.33 N)	208.33 g (2.04 N)	274.00 g (2.69 N)	343.33 g (3.36 N)
LMB third finger	77 g (0.75 N)	175 g (1.72 N)	282 g (2.76 N)	402 g (3.94 N)	532 g (5.22 N)
LMB fourth finger	57 g (0.56 N)	142 g (1.39 N)	245 g (2.40 N)	367 g (3.60 N)	460 g (4.51 N)
LMB fifth finger	117 g (1.15 N)	207 g (2.03 N)	310 g (3.04 N)	397 g (3.89 N)	520 g (5.10 N)
MEAN LMB	83.67 g (0.82 N)	174.67 g (1.81 N)	279.00 g (2.74 N)	388.67 g (3.81 N)	504.00 g (4.94 N)

**Table 2:** Mean Forces of different ETDNO made with tubes at different angled positions. We considered the forces in bold to be within the therapeutic range.

	0°	15°	30°	45°	60°
Hyperextension	102 g (1.00 N)	195 g (1.91 N)	250 g (2.45 N)	320 g (3.14 N)	432 g (4.24 N)
0°	50 g (0.49 N)	140 g (1.37 N)	238 g (2.33 N)	310 g (3.04 N)	390 g (3.82 N)
15	0 g	97 g (0.95 N)	170 g (1.67 N)	225 g (2.21 N)	315 g (3.09 N)
30	0 g	60 g (0.59 N)	127 g (1.24 N)	195 g (1.91 N)	260 g (2.55 N)
45	0 g	17 g (0.17 N)	95 g (0.93 N)	157 g (1.54 N)	237 g (2.32 N)

**Table 3:** Contact pattern of straight tube ETDNO, flexed tube ETDNO and LMB orthosis.

	ETDNO 0° at 0°	ETDNO 0° at 30°	ETDNO 30° at 30°	LMB at 0°	LMB a 30°
Total finger surface	51 cm^2^	51 cm^2^	51 cm^2^	51 cm^2^	51 cm^2^
Potential volar contact surface	14.875 cm^2^	14.875 cm^2^	14.93 cm^2^	14.875 cm^2^	14.875 cm^2^
Potential dorsal contact surface	14.875 cm^2^	14.875 cm^2^	16.69 cm^2^	14.875 cm^2^	14.875 cm^2^
Volar contact surface	14.875 cm^2^	14.875 cm^2^	14.93 cm^2^	5.7 cm^2^	5.4 cm^2^
% volar contact	100%	100%	100%	38.3%	36.3%
Dorsal contact surface	12.32 cm^2^	10.41 cm^2^	15.49 cm^2^	2.6 cm^2^	2.4 cm^2^
% dorsal contact	83%	70%	93%	17.5%	16%

**Table 4:** Orthosis forces, contact surfaces and pressure pattern on straight tube ETDNO, 30° flexed tube ETDNO and LMB 501 orthosis.

	ETDNO 0° at 0°	ETDNO 0° at 30°	ETDNO 30° at 30°	LMB at 0°	LMB a 30°
Mean distal force	50 g (0.49 N)	238 g (2.33 N)	170 g (1.67 N)	77 g (0.75 N)	282 g (2.76 N)
Volar contact surface	14.875 cm^2^	14.875 cm^2^	14.93 cm^2^	5.7 cm^2^	5.4 cm^2^
Volar pressure in g/cm^2^	7.79	28	22.7	28.6	104.4
Volar pressure mmHG	5.73	20.59	16.69	21.037	76.79
Mean force at dorsal PIPJ	100	459.8	328.41	154	544.78
Dorsal contact surface	12.32	10.41	15.49	2.6	2.4
Dorsal pressure g/cm^2^	8.11	44.09	15.39	59.23	226.99
Volar pressure mmHG	5.97	32.43	11.32	43.57	166.96

## Data Availability

Not applicable.

## Supplementary Materials

The following supporting information can be downloaded at: https://www.mdpi.com/article/10.3390/jcm12082855/s1; Table S1: Differences between LMB and ETDNO in the third finger; Table S2: Differences between LMB and ETDNO in the fourth finger; Table S3: Differences between LMB and ETDNO in the fifth finger.
