# Supplementary material for: A Cadaver Based Comparison of Two Elastic Tension Proximal Interphalangeal Joint (PIPJ) Extension Orthoses with Focus on Force Generation and Pressure Distribution
Source: J Clin Med. 2023 Apr 13;12(8):2855. doi: 10.3390/jcm12082855 (PMC10145399; doi:10.3390/jcm12082855)
Supplement: Supplementary file 1 [file jcm-12-02855-s001.zip › jcm-2245620-supplementary.pdf]

Journal of
Clinical Medicine

MDPI

Supplementary material

Our results show that there are significant differences between all the compared values of ETDNO and LMB except in ETDNO D3 hyperextension 15º and LMB 15º. This means that the forces that make LMB 15º are equivalent to those made by an ETDNO D3 15º in hyperextension. The comparison of D4, LMB med and SL show that there are differences between all splints except between LMB med 0º and LMB SL 0º and ETDNO D4 0º. So these splints are equivalent in strength to 0º.

Supplementary Table S1. Differences between LMB and ETDNO in the third finger. Values marked with * are statistically significant.

**LMB and ETDNO in the third finger**	*LMB 0 º*	*LMB 15º*	*LMB 30º*	*LMB 45º*	*LMB 60º*
ETDNO D3 straight 0º	Yes<br>U= 10<br>P= 0.002*	-	-	-	-
ETDNO D3 hyperextension 0º	Yes<br>U= 14<br>P= 0.005*	-	-	-	-
ETDNO D3 15º at 0º	Yes<br>U= 0<br>P< 0.0001*	-	-	-	-
ETDNO D3 30 º at 0º	Yes<br>U= 0<br>P< 0.0001*	-	-	-	-
ETDNO D3 45 º at 0º	Yes<br>U= 0<br>P< 0.0001*	-	-	-	-
ETDNO D3 straight 15º	-	Yes<br>U= 9<br>P= 0.001*	-	-	-
ETDNO D3 hyperextension 15º	-	Yes<br>U= 23<br>P= 0.04*	-	-	-
ETDNO D3 15 º at 15º	-	Yes<br>U= 0<br>P< 0.0001*	-	-	-
ETDNO D3 30 º at 15º	-	Yes<br>U= 0<br>P< 0.0001*	-	-	-
ETDNO D3 45 º at 15 º	-	Yes<br>U= 0<br>P< 0.0001*	-	-	-
ETDNO D3 straight 30º	-	-	Yes<br>U= 7.5<br>P< 0.0001*	-	-
ETDNO D3 hyperextension 30º	-	-	Yes<br>U= 19.5<br>P= 0.02*	-	-
ETDNO D3 15 º at 30º	-	-	Yes<br>U= 0<br>P< 0.0001*	-	-
ETDNO D3 30 º at 30º	-	-	Yes<br>U= 0<br>P< 0.0001*	-	-
ETDNO D3 45º at 30º	-	-	Yes<br>U= 0<br>P< 0.0001*	-	-
ETDNO D3 straight 45 º	-	-	-	Yes<br>U= 0<br>P< 0.0001*	-
ETDNO D3 hyperextension 45 º	-	-	-	Yes<br>U= 1<br>P< 0.0001*	-
ETDNO D3 15 º at 45º	-	-	-	Yes<br>U= 0	-

Supplementary Table S2. Differences between LMB and ETDNO in the fourth finger

**LMB and ETDNO in the fourth finger**	*LMB med 0 º*	*LMB SL 0º*	*LMB med 15º*	*LMB SL 15º*	*LMB med 30º*	*MB SL 30º*	*LMB med 45º*	*LMB SL 45º*	*LMB med 60º*	*LMB SL 60º*
ETDNO D4 0º	No U= 32 P= 0.19	No U= 44 P= 0.68	-	-	-	-	-	-	-	-
ETDNO D4 15º	-	-	Yes U= 11.5 P= 0.002*	No U= 36 P= 0.31	-	-	-	-	-	-
ETDNO D4 30º	-	-	-	-	Yes U= 0.5 P< 0.0001*	Yes U= 23 P= 0.04*	-	-	-	-
ETDNO D4 45º	-	-	-	-	-	-	Yes U= 0 P< 0.0001*	Yes U= 7 P< 0.0001*	-	-
ETDNO D4 60º	Yes U= 0 P< 0.0001*	-	-	-	-	-	-	-	Yes U= 0 P< 0.0001*	Yes U= 3 P< 0.0001*

Supplementary Table S3. Differences between LMB and ETDNO in the fifth finger

**LMB and ETDNO in the fifth finger**	*LMB 0 º*	*LMB 15º*	*LMB 30º*	*LMB 45º*	*LMB 60º*
ETDNO D5 0º	Yes<br>U=12.5<br>P=0.03	-	-	-	-
ETDNO D5 15º	-	Yes<br>U=6<br>P< 0.0001*	-	-	-
ETDNO D5 30º	-	-	Yes<br>U=5<br>P< 0.0001*	-	-
ETDNO D5 45º	-	-	-	Yes<br>U=1<br>P< 0.0001*	-
ETDNO D5 60º	-	-	-	-	Yes<br>U=0<br>P< 0.0001*
